# Virologic Response to Tipranavir-Ritonavir or Darunavir-Ritonavir Based Regimens in Antiretroviral Therapy Experienced HIV-1 Patients: A Meta-Analysis and Meta-Regression of Randomized Controlled Clinical Trials

**DOI:** 10.1371/journal.pone.0060814

**Published:** 2013-04-04

**Authors:** Asres Berhan, Yifru Berhan

**Affiliations:** Hawassa University College of Medicine and Health Sciences, Hawassa, Ethiopia; Alberta Provincial Laboratory for Public Health, University of Alberta, Canada

## Abstract

**Background:**

The development of tipranavir and darunavir, second generation non-peptidic HIV protease inhibitors, with marked improved resistance profiles, has opened a new perspective on the treatment of antiretroviral therapy (ART) experienced HIV patients with poor viral load control. The aim of this study was to determine the virologic response in ART experienced patients to tipranavir-ritonavir and darunavir-ritonavir based regimens.

**Methods and Findings:**

A computer based literature search was conducted in the databases of HINARI (Health InterNetwork Access to Research Initiative), Medline and Cochrane library. Meta-analysis was performed by including randomized controlled studies that were conducted in ART experienced patients with plasma viral load above 1,000 copies HIV RNA/ml. The odds ratios and 95% confidence intervals (CI) for viral loads of <50 copies and <400 copies HIV RNA/ml at the end of the intervention were determined by the random effects model. Meta-regression, sensitivity analysis and funnel plots were done. The number of HIV-1 patients who were on either a tipranavir-ritonavir or darunavir-ritonavir based regimen and achieved viral load less than 50 copies HIV RNA/ml was significantly higher (overall OR = 3.4; 95% CI, 2.61– 4.52) than the number of HIV-1 patients who were on investigator selected boosted comparator HIV-1 protease inhibitors (CPIs-ritonavir). Similarly, the number of patients with viral load less than 400 copies HIV RNA/ml was significantly higher in either the tipranavir-ritonavir or darunavir-ritonavir based regimen treated group (overall OR = 3.0; 95% CI, 2.15 – 4.11). Meta-regression showed that the viral load reduction was independent of baseline viral load, baseline CD4 count and duration of tipranavir-ritonavir or darunavir-ritonavir based regimen.

**Conclusions:**

Tipranavir and darunavir based regimens were more effective in patients who were ART experienced and had poor viral load control. Further studies are required to determine their consistent viral load suppression effect as the duration of treatment is more prolonged.

## Introduction

The advent of ART in 1995 has added 14 million life-years for HIV infected people in low- and middle-income countries [1]. These days, the widely used ART regimen in HIV infected individuals is a combination of three drugs; two nucleoside reverse transcriptase inhibitors (NRTIs) and one non-nucleoside reverse transcriptase inhibitor (NNRTI) or one protease inhibitor (PI) alone or with ritonavir (booster). Despite the pills burden, the main challenge in the treatment of HIV-1 infection is the emergence of drug resistant HIV strains. In six years of ART, the risk of mutations to at least two of the three main drug classes was about 20% [2]. The transmission of drug resistant HIV from an index case to others is also becoming more frequent [3]. It has been shown that once resistance develops to one of antiretroviral drugs, the chance of becoming resistant to other antiretroviral drugs (cross resistance) is very high.

As compared to other classes of antiretroviral drugs, PIs have the highest genetic barrier to resistance [4]. Nevertheless, time to develop resistance to non-boosted PI based regimens (without ritonavir) was not different from NNRTI based regimens. However, the time to develop resistance to boosted PIs based regimens (with ritonavir) was greatly reduced [5]. The delay for emergence of resistance in ritonavir boosted PIs was attributed to its inhibition effect on the cytochrome P450 mediated metabolism of other PIs [6]. So far, however, it was noted that the extensive use of first generation PIs has led to the emergence of PI resistant HIV-1 strains, which are cross resistant to most members of this class [4], [7], [8].

On the other hand, the development of tipranavir and darunavir, second generation non-peptidic HIV-1 PIs with markedly improved resistance profiles, has opened a new perspective in the treatment of ART experienced patients with poor viral control or developed resistant HIV strains. Patients who received tipranavir-ritonavir (TPV/r) with an optimized background regimen have achieved and maintained more treatment response than patients who received investigator selected comparator PIs (CPIs) with optimized background regimens [9]–[11]. Similarly, patients who received darunavir-ritonavir (DRV/r) with optimized background regimens also achieved and maintained more treatment response than patients who received CPIs-ritonavir based regimens [12]–[18].

Even though there are several studies on both tipranavir and darunavir, to the best of the authors’ knowledge, no published meta-analysis has assessed their consistent efficacy in ART experienced HIV patients with poor viral control. Thus, the primary aim of this meta-analysis was to determine the virologic response to tipranavir-ritonavir (500 mg/200 mg) twice daily and darunavir-ritonavir (600 mg/100 mg) twice daily based regimens, as compared to CPIs based regimens in ART experienced HIV-1 patients.

## Methods

### Search strategy

A computer based literature search was conducted in the databases of HINARI, Medline and the Cochrane library. The search was further strengthened by searching literature using Google scholar search engine and literature from the reference lists of retrieved articles. Our search terms included: “tipranavir”, “PNU-140690”, “darunavir”, “TMC114”, “second generation PI” and “virologic response”. During searching, the search terms were combined alternatively with Boolean logic (and/or).

### Inclusion criteria and study selection

In this meta-analysis, the pre-determined inclusion criteria were: 1) randomized controlled studies that assessed the effectiveness of tipranavir-ritonavir (500 mg/200 mg) twice daily or darunavir-ritonavir (600 mg/100 mg) twice daily based regimens relative to investigator selected boosted CPIs in ART experienced HIV-1 patients; 2) studies that recruited HIV type 1 patients with plasma viral load above 1,000 copies HIV RNA/ml; and 3) studies conducted in English. These criteria were set to address the primary aim of this meta-analysis, which was to determine the effectiveness of tipranavir and darunavir in ART experienced HIV-1 patients (probably with resistant HIV strains) as compared to CPIs and the authors’ inability to translate articles written in other languages.

Study selection was performed in two stages. Firstly, the titles and abstracts of all the retrieved articles were reviewed and then grouped as “eligible for inclusion” and “ineligible for inclusion”. Secondly, articles which were grouped under “eligible for inclusion” were reviewed in detail for decision.

### Data extraction

For data extraction, a standard Excel spreadsheet was used; and data were abstracted individually. From the included studies, the following data were extracted: the first author’s name, year of publication, names of the PIs that were given in each group in the study period, duration of therapy, frequency of drug administration, strength of the drugs (dose), sample size in each group, baseline viral load, baseline CD4 count, number of patients who achieved viral load reduction by ≥1 log_10_ copies HIV RNA/ml from the baseline in each group, number of patients with viral load <400 copies HIV RNA/ml in each group at the end of interventions, and number of patients with viral load <50 copies HIV RNA/ml in each group at the end of interventions.

### Operational definitions

In this meta-analysis the term “ART experienced” means a patient who was on anti-HIV treatment (usually including a combination of three or more antiretroviral drugs) with poor viral load control (viral load >1000 copies HIV RNA/ml). “Investigator selected boosted comparator protease inhibitors” (CPIs) – means any of the currently available first generation HIV PIs with ritonavir as a booster (CPI-r).

### Statistical analysis

Meta-analysis was carried out to assess the efficacy of tipranavir-ritonavir and darunavir-ritonavir plus an optimized background regimen relative to ritonavir boosted CPIs in ART experienced HIV patients. The odds ratios (OR) and the 95% CIs for: (a) achieved a viral load reduction by ≥1 log_10_ copies HIV RNA/ml from the baseline, (b) viral load <400 copies HIV RNA/ml at the end of the interventions, and (c) viral load <50 copies HIV RNA/ml load at the end interventions were computed with the DerSimonian-Laird method (random effects model).

The heterogeneity among the studies was assessed by computing values for chi-square (Q), I^2^ and p-values. I^2^ ≥50% was considered as statistically significant. When there was significant heterogeneity, subgroup analysis and meta-regression analysis were conducted to determine the possible contributing factors for the heterogeneity. Meta-regressions were conducted based on the duration of therapy, baseline viral load and baseline CD4 count. Sensitivity analyses (leaving one study out at a time) were also conducted to estimate the stability of the overall odds ratios in the withdrawal of any of the studies from the analysis. Publication/disclosure biases were assessed with funnel plots. All the meta-analyses and plots were generated using Review Manager (RevMan) Version 5.1 software. Comprehensive Meta-Analysis Software was used to do meta-regression.

## Results

**Figure 1:**
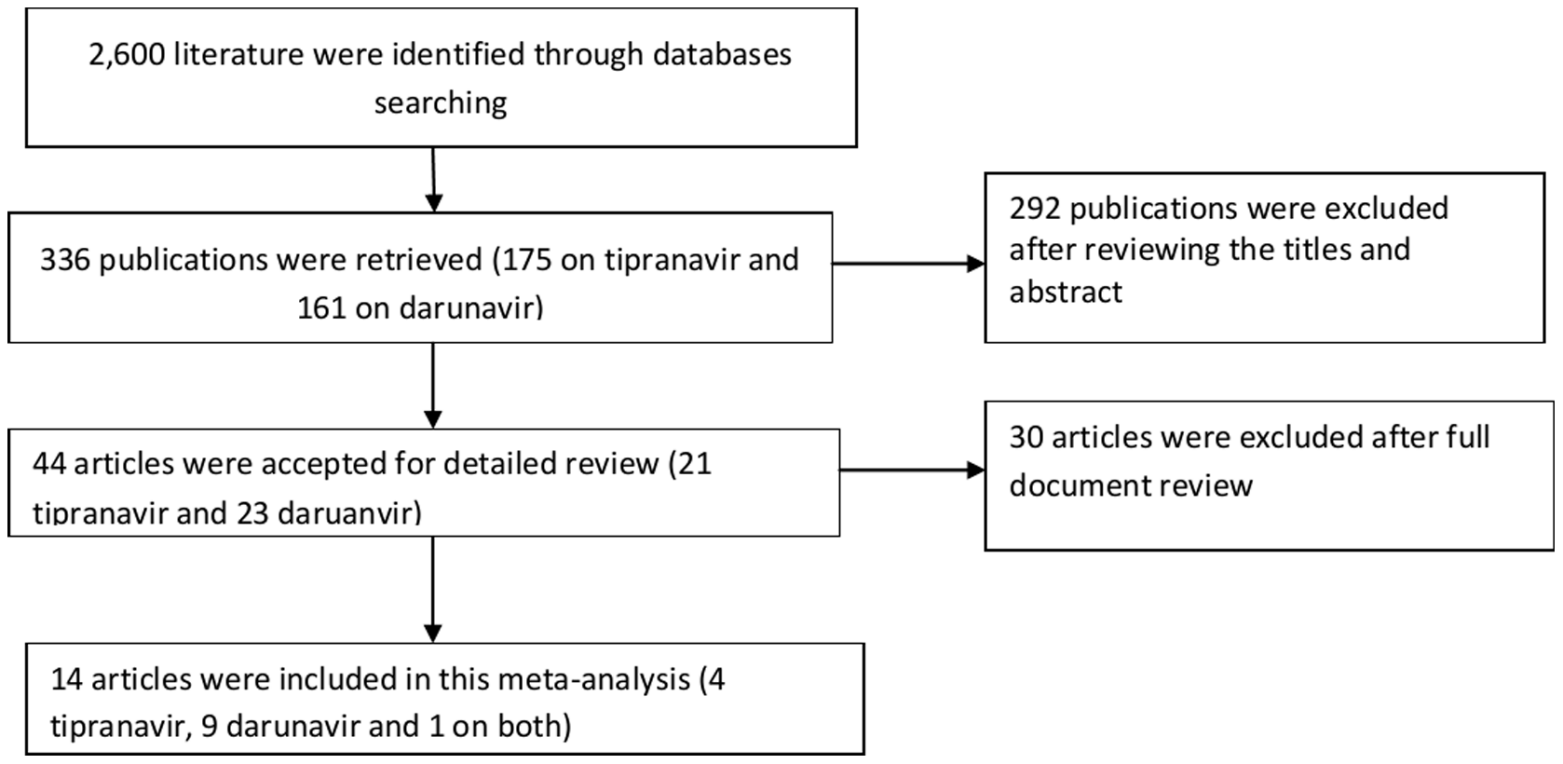
Flow diagram showing studies selection.

As presented in Fig 1, from the retrieved 336 publications on tipranavir-ritonavir and darunavir-ritonavir, only fourteen articles met the predefined inclusion criteria. Of these, 4 were on tipranavir [9]–[11], [19], 9 were on darunavir [12]–, [20], [21], and 1 was on both tipranavir and darunavir [22]. From the included studies, 11 were exclusively conducted on HIV-1 patients with one or more primary PI-associated mutations [9]–[11], [14], [16]–[22]. In general, funnel plots did not demonstrate the existence of publication/disclosure biases.

**Figure 2:**
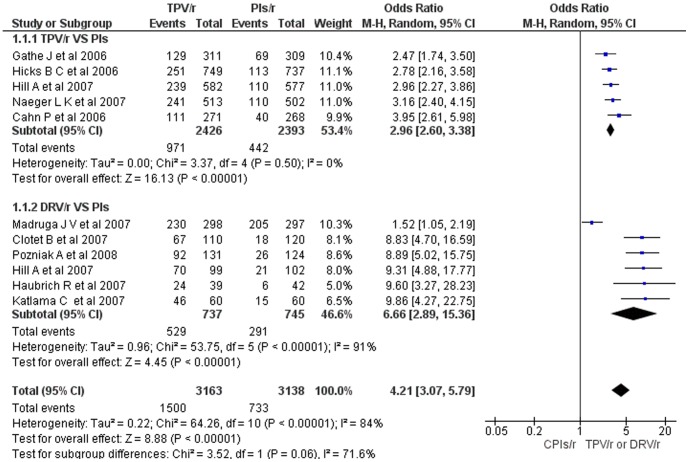
Mantel-Haenszel odds ratio of ART experienced HIV-1 patients with HIV-1 RNA reduction ≥1 log_10_ copies/ml from baseline (TPV/r or DRV/r vs CPIs/r).

The meta-analysis (including 3,163 HIV-1 patients who were on either tipranavir-ritonavir or darunavir-ritonavir based regimens and 3,138 HIV-1 patients who were on CPIs-ritonavir based regimens) of viral load reduction by ≥1 log_10_ copies HIV RNA/ml from the baseline showed a statistically significant reduction in patients who were on either tipranavir-ritonavir or darunavir-ritonavir based regimens (overall OR = 4.2; 95% CI, 3.07 – 5.79) (Fig 2). As compared to boosted CPIs treated, the viral load reduction in the subgroup of darunavir-ritonavir treated was larger (OR = 6.66) than the viral load reduction in tipranavir-ritonavir treated (OR = 2.96). Heterogeneity testing revealed a statistically significant variation among the studies which compared darunavir-ritonavir based regimens with boosted CPIs based regimens (I^2^ = 91%) but no significant heterogeneity among the studies that compared tipranavir-ritonavir based regimens with boosted CPIs based regimens (I^2^ = 0%). In the sensitivity analysis, the overall OR swings between 3.9 and 4.6; however, when Madruga JV et al [15] was withdrawn from the analysis, the heterogeneity among all the included studies and in the subgroup of boosted darunavir treated was not significant.

**Figure 3:**
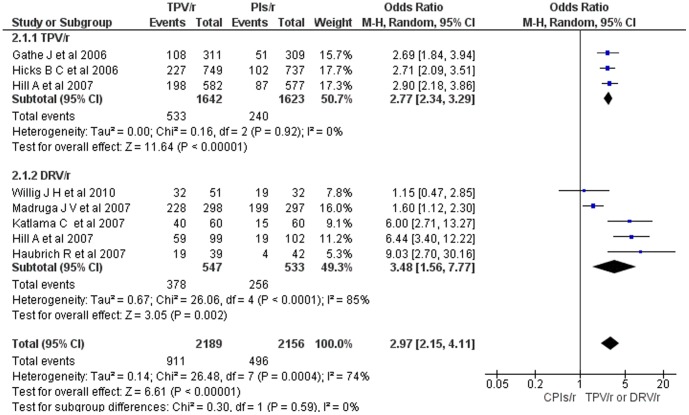
Mantel-Haenszel odds ratio of ART experienced HIV-1 patients who achieved HIV-1 RNA < 400 copies per ml (TPV/r or DRV/r vs CPIs/r).

The meta-analysis of HIV-1 patients who achieved a viral load below 400 copies of HIV RNA/ml at the end of the interventions was conducted by including 2,189 HIV-1 patients who were on either tipranavir-ritonavir or darunavir-ritonavir based regimens and 2,156 HIV-1 patients who were on CPIs-ritonavir based regimens. In the tipranavir-ritonavir or darunavir-ritonavir based regimen treated group, in all studies except for that of Willing JH et al [12], the number of patients who achieved viral load less than 400 copies HIV RNA/ml was significantly higher (overall OR = 3.0; 95% CI, 2.15 – 4.11) (Fig 3). Sensitivity analysis showed that the overall OR changed by about 0.3 at maximum with the withdrawal of any of the included studies. The odds of patients achieving a viral load less than 400 copies HIV RNA/ml in the darunavir-ritonavir treated and tipranavir-ritonavir based regimen treated subgroups relative to CPIs-ritonavir treated were about 3.5 and 2.8 times higher, respectively. Unlike studies on tipranavir, heterogeneity testing still showed a significant inconsistency among studies on darunavir. Nevertheless, when the studies on darunavir were subgrouped as: 1) studies only on patients with primary PI-associated mutation, and 2) studies that were not necessarily on patients with primary PI-associated mutation, the heterogeneity testing showed no significant variability.

**Figure 4:**
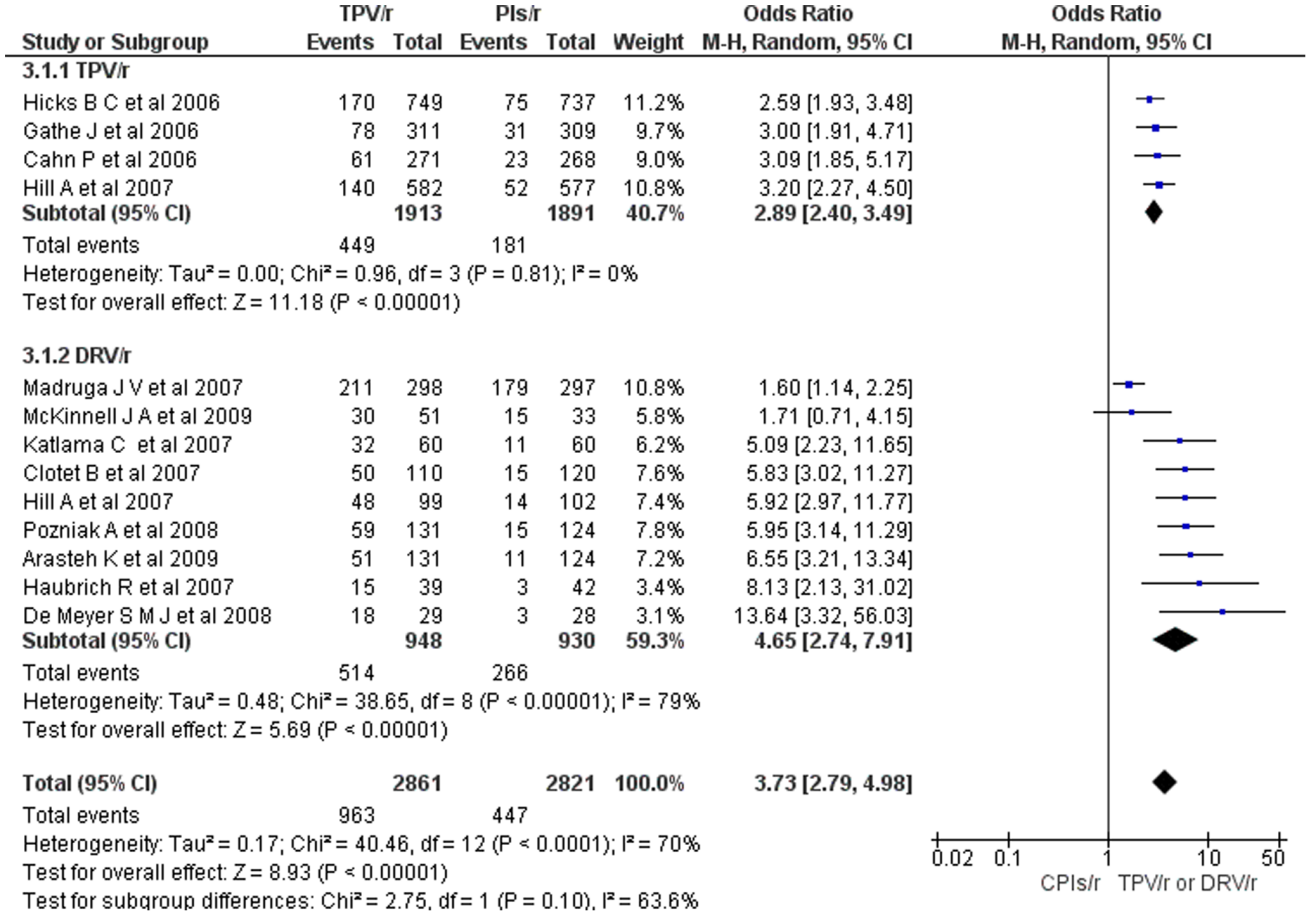
Mantel-Haenszel odds ratio of ART experienced HIV-1 patients who achieved HIV-1 RNA < 50 copies per ml (TPV/r or DRV/r vs CPIs/r).

Similarly, the meta-analysis including 2,861 HIV-1 patients who were on either tipranavir-ritonavir or darunavir-ritonavir based regimens and 2,821 HIV-1 patients who were on CPIs-ritonavir based regimens, the number of HIV-1 patients who achieved a viral load less than 50 copies HIV RNA/ml at the end of the interventions was significantly higher in patients who were on either tipranavir-ritonavir or darunavir-ritonavir based regimens (overall OR = 3.4; 95% CI, 2.61– 4.52) (Fig 4.). All the included studies on tipranavir (OR = 2.9; 95% CI, 2.40 – 3.49) and seven of the nine studies on darunavir (OR = 3.9; 95% CI, 2.36 – 6.46) showed a statistically significant viral load reduction. However, heterogeneity testing yet again affirmed the significant variability of studies on darunavir (I^2^ = 79%, Q = 38.65, P<0.00001) and the uniformity of studies on tipranavir.

However, the significant heterogeneity among studies on darunavir was largely due to Madruga JV et al [15] (in this study, PI-associated mutations was not put as HIV patients eligibility criteria and compared darunavir-ritonavir with that of lopinavir-ritonavir in treatment-experienced lopinavir-naive patients); after partitioning for this study, there was no significant residual heterogeneity (I^2^ = 22%, Q = 9.00, P = 0.25).

Meta-regressions were conducted to determine how the duration of tipranavir-ritonavir based therapy, baseline viral load and baseline CD4 count affects the reduction of viral load by ≥1 log_10_ copies HIV RNA/ml from the baseline, the number of patients who achieved viral load less than 400 copies HIV RNA/ml and number of patients who achieved viral load less than 50 copies at the end of the interventions. But, the computed values revealed the lack of relation between the efficacy of tipranavir-ritonavir or darunavir-ritonavir based regimens and the duration of therapy, the baseline viral load and baseline CD4 count (Table 1). In other words, the viral suppression effect of boosted tipranavir or darunavir based regimens was not dependent on the duration of therapy, the initial viral load and the CD4 count at the start of interventions.

**Table 1:** Summary of findings from meta-regression analyses.

Variable	Regression coefficient	Standard error	95% CI	p-value
Effect of duration of therapy on:				
HIV-1 RNA reduction ≥1 log_10_ copies/ml from baseline	–0.01252	0.01505	–0.04202, 0.01699	0.40563
Achievement of HIV-1 RNA <400 copies/ml	–0.02405	0.01318	–0.04989, 0.00179	0.06807
Achievement of HIV-1 RNA <50 copies/ml	0.00589	0.00759	–0.00898, 0.02076	0.4370
Effect of baseline viral load (log copies/ml) on:				
HIV-1 RNA reduction ≥1 log_10_ copies/ml from baseline	–0.19182	2.51874	–5.1845, 4.74481	0.93929
Achievement of HIV-1 RNA <50 copies/ml	1.36139	1.10178	–0.79805, 3.52038	0.21660
Effect of baseline CD4 count (cells/ml) on:				
HIV-1 RNA reduction ≥1 log_10_ copies/ml from baseline	–0.01045	0.01171	–0.03339, 0.01250	0.37223
Achievement of HIV-1 RNA <400 copies/ml	–0.00369	0.01790	–0.03876, 0.03139	0.83681
Achievement of HIV-1 RNA <50 copies/ml	0.00995	0.00533	–0.02039, 0.00049	0.06183

## Discussion

This meta-analysis demonstrated that treatment with tipranavir-ritonavir or darunavir-ritonavir based regimen in ART experienced HIV-1 patients showed a statistically significant viral load reduction. In other words, though all patients included in the selected studies had a plasma viral load above 1,000 copies HIV RNA/ml at the time of recruitment, the number of patients who achieved a viral load of <50 copies HIV RNA/ml was significantly higher in the group of tipranavir-ritonavir or darunavir-ritonavir based regimens treated than in the CPIs-ritonavir based regimens treated group. Such an extent of viral load suppression with tipranavir-ritonavir or darunavir-ritonavir based regimens in ART experienced is an advantage to meet the ART guideline recommendation of making the viral load below 400 copies HIV RNA/ml within six months [23]. For the best, it is recognized that patients who were on ART and achieved viral load of <50 copies HIV RNA/ml had longer responses as compared to patients who achieved viral load of 50–400 copies HIV RNA/ml [24], [25].

Specifically, relative to CPIs-ritonavir based regimens, the odds of viral load suppression of darunavir-ritonavir based regimens were greater than the odds of tipranavir-ritonavir based regimes. However, studies that directly compared tipranavir-ritonavir based regimens with darunavir-ritonavir based regimens did not show significant difference in terms of virologic suppression [26], [27]. In this analysis, even though the viral load suppression of darunavir-ritonavir based regimens was significantly higher in studies where primary PI-associated mutations was not put as HIV patients’ eligibility criteria [12], [13], [15], it was not as high as other studies that put primary PI-associated mutations as an eligibility criteria [14], [16]–[22]. In other words, the viral load suppression of darunavir-ritonavir based regimens was higher in patients with one or more primary PI-associated mutations as compared to CPIs-ritonavir based regimens. Similarly, though all the studies were on HIV-1 patients with one or more primary PI-associated mutations, all the included studies on tipranavir-ritonavir based regimens showed a significant viral load reduction. Thus, this finding reaffirmed that tipranavir and darunavir based regimens were more effective in patients who were ART experienced and had resistant HIV-1 strains.

Other primary studies that compared the effectiveness of darunavir-ritonavir based regimens with lopinavir-ritonavir based regimens on ART naïve HIV patients also showed a significantly greater viral load reduction effect of darunavir-ritonavir based regimens [28], [29]. The observed significant viral load reduction in ART naïve and ART experienced HIV patients (with or without one or more primary PI-associated mutations) in tipranavir and darunavir based regimens is attributed to their structural difference from other PIs. This is to say; darunavir and tipranavir binds to the active site of the HIV protease enzyme with fewer hydrogen bonds than other PIs. This mechanism allows for increased flexibility of tipranavir and darunavir adjusting the HIV mutations in the active site [30], [31]. Additionally, darunavir has a higher binding affinity with wild-type of protease enzyme and has a higher dissociative half-life than other PIs [32].

Heterogeneity testing has shown the existence of significant variability among the included studies on darunavir. However, as the meta-regression showed, the variation in duration of therapy, baseline viral load and CD4 count among the included studies were not adequate to explain the sources or causes of heterogeneity. However, the lack of relation between the duration of tipranavir-ritonavir or darunavir-ritonavir based therapy and viral load reduction should be interpreted cautiously, because a Taiwanese study with a longer duration of therapy than any of the studies included in this meta-analysis showed a significant increase in the levels of genotypic resistance to tipranavir and darunavir in patients with virological failure to first generation PIs [33]. Thus, the most likely explanation for the heterogeneity on darunavir is the variation of HIV patients recruited in the included studies on the HIV’s PI-associated mutations; three of the eleven included studies on darunavir did not require an inclusion criterion of HIV-1 patients with one or more primary PI-associated mutations. Another likely explanation is the variation in the type of antiretroviral drugs and the duration of therapy used in the previous treatment. A study has also shown the correlation of tipranavir resistance with the type of first generation PIs and duration of therapy in the previous exposure [34].

As limitations of this analysis, firstly, since most of the included studies did not report the types of drugs and the duration of therapies in the previous PI based regimens, this analysis was not able to assess their impacts on the therapeutic outcomes of tipranavir-ritonavir and darunavir-ritonavir based regimens. Secondly, the available data on CD4 counts were incomplete, making it difficult to determine the effects of tipranavir and darunavir based regimens on changes in CD4 count. However, based on previous studies that found an inverse relation of CD4 count and plasma viral load [35], [36], the significant viral load reduction in patients who were on tipranavir-ritonavir or darunavir-ritonavir based regimens is assumed to be accompanied by a significant CD4 count increment. Thirdly, although the funnel plots did not support disclosure biases, all the included studies were sponsored by pharmaceutical companies, raising the possibility of biases by comparator selection to get significant effect of the investigational drugs and the selection for publication as has been pointed out by other investigators [37]–[39].

In conclusion, this meta-analysis has shown a significant viral load reduction in ART experienced HIV-1 patients who were treated with tipranavir-ritonavir or darunavir-ritonavir based regimens as compared to CPIs-ritonavir based regimens. Furthermore, the viral load reduction was independent of baseline viral load, baseline CD4 count and duration of tipranavir-ritonavir or darunavir-ritonavir based regimens. However, further studies are required to determine their consistent plasma viral load suppression effect as the duration of treatment is more prolonged.
